# Value of focused lung ultrasound in diagnosing and monitoring pneumothorax after CT-guided lung biopsy

**DOI:** 10.1007/s40477-025-00998-w

**Published:** 2025-02-27

**Authors:** Emma Katrine Metzler, Nina Everløff, Amanda Dandanell Juul, Christian Borbjerg Laursen, Ole Graumann, Pia Iben Pietersen

**Affiliations:** 1 Department of Radiology, Odense University Hospital, Odense, Denmark; 2 Department of Radiology, Sygehus Lillebaelt, Vejle, Denmark; 3 Department of Internal Medicine, Odense University Hospital Svendborg, Svendborg, Denmark; 4 Odense Respiratory Research Unit (ODIN), Department of Clinical Research, University of Southern Denmark, Odense, Denmark; 5 Department of Respiratory Medicine, Odense University Hospital, Odense, Denmark; 6 Department of Radiology, Aarhus University Hospital, Aarhus, Denmark; 7 Present Address: UNIFY, Research and Innovation Unit of Radiology, University of Southern Denmark, Odense, Denmark

**Keywords:** Thoracic ultrasound, Pneumothorax, Monitoring post-biopsy, Cancer, CT-guided transthoracic lung biopsy

## Abstract

**Background:**

Pneumothorax is a common complication after CT-guided transthoracic lung biopsy. Pneumothorax is most often diagnosed with a conventional chest X-ray after a two-hour observation period. Ultrasound has been shown to be superior to conventional X-rays in diagnosing pneumothorax in some settings and it can be repeated without radiation exposure.

**Purpose:**

The aim of the study was firstly to explore the sensitivity and specificity of thoracic ultrasound compared to conventional chest x-ray following CT-guided transthoracic lung biopsy, and secondly to investigate the dynamic changes of pneumothorax size using ultrasound.

**Methods:**

This prospective study was conducted at Odense University Hospital in the Department of Radiology. Adult patients undergoing CT-guided lung biopsy were eligible for inclusion. A total of 26 patients were included in the study and were scanned five times in sitting upright and supine positions during the two-hour observation time using the FLUS Protocol.

**Results:**

Pneumothorax was diagnosed via chest x-ray in 11 (42%) patients. With the patient in an upright position, the ultrasound had a sensitivity of 63.6% and a specificity of 93.3%. These numbers rose to 72.3% and 100% in the supine position. Monitoring the pneumothoraces showed a slight increase in size during the two hours.

**Conclusions:**

Ultrasound can be used to diagnose pneumothorax after CT-guided lung biopsy. All pneumothoraxes that were identified by ultrasound were detected within the first 30 min. The dynamic changes of pneumothoraxes showed that the size of the pneumothorax did not increase to a level where the patient needed intervention or admittance.

## Introduction

Lung cancer, the leading cause of cancer-related deaths worldwide, claims approximately 1.8 million lives each year. The diagnostic process for suspected lung cancer relies heavily on cytopathological and molecular studies of the cancer-suspect tissue which is often acquired through endobronchial or CT-guided transthoracic biopsy [1–4]. Despite its overall safety, the procedure of CT-guided transthoracic lung biopsy carries a known risk of complications with pneumothorax, the focus of this study, being one of the most frequent [3, 5, 6].

Typically, patients undergoing CT-guided transthoracic needle biopsy are observed and monitored for a few hours afterwards for potential complications, with chest X-ray serving as the standard imaging modality for detecting complications including pneumothorax. However, emerging evidence suggests that thoracic ultrasound specifically of the lungs (FLUS) has shown high diagnostic accuracy for many of the common causes of respiratory distress, including pneumothorax – therefore, FLUS has gained substantial attention, and its use has increased drastically within the last decade in various settings: from the emergency department to the intensive care unit, in the respiratory outpatient clinic and prehospital [7–9]. LUS is a relatively fast ultrasound examination that provides the operator with answers to specific clinical questions, can be performed at the bedside without pain, and with no radiation exposure.

Very little is explored about lung ultrasound and its diagnostic performance in a post-CT-guided transthoracic lung biopsy procedure where the prevalence of pneumothorax is potentially higher as well as the use of lung ultrasound for repeated monitoring pneumothorax sizes in this setting [10, 11]. One study even proposed that FLUS could be superior to chest X-ray in detecting pneumothorax following biopsies, providing a quicker and potentially more accurate diagnosis [11].

Although the diagnosis, risk factors, and management of pneumothorax have been widely studied [4, 12–16], less attention has been given to ongoing monitoring of pneumothoraces over time [10, 17, 18]. Recent guidelines and recommendations on the management and treatment of spontaneous pneumothorax have favoured a more conservative approach, emphasizing the potential of ultrasound monitoring of pneumothorax size over time, and potentially transferring the pending and conservative approach to the iatrogenic pneumothorax in the future.

The primary aim of the study was to explore the sensitivity and specificity of thoracic ultrasound in a radiological outpatient clinic setting following a CT-guided transthoracic biopsy procedure, and, secondly, to investigate how ultrasound performs as a monitoring tool for the dynamic changes of pneumothorax size over time.

## Methods

### Study design and setting

This observational study was conducted at the Department of Radiology, Odense University Hospital from April to December 2021 and reported according to STROBE guidelines [19]. The study protocol was submitted to the Regional Committees on Health Research Ethics for Southern Denmark (ID: 21/18582) and the application was unnecessary. Patient data was handled in accordance with the Danish Data Protection Act.

### Participants

Patients aged 18 or older with suspected pathological lung lesions undergoing CT-guided lung biopsy at Odense University Hospital were eligible to enter the study. Patients were excluded if the biopsy was not performed, for example, due to patient non-compliance or unreachable tumours. If patients had the biopsy and developed complications needing urgent intervention, e.g., large symptomatic pneumothoraces needing drainage, the patients were transferred to the Department of Respiratory Medicine for hospitalisation and thereby, were not included in the study. The patients could be included more than once if they for any reason needed another biopsy, this counting as a separate patient entry in the study. Each patient was informed orally and written about the project by the ultrasound operators before being given the option to consent or refuse participation.

### CT-guided procedure

One of three interventional radiologists with at least two years of experience and between 100 – 1.500 procedures performed the CT-guided transthoracic needle biopsy. All procedures were performed in local anaesthesia. The procedure was guided using a Siemens Somatom Flash CT scanner (Siemens Healthcare GmbH, Erlangen, DE). The biopsies were performed as core needle biopsies using an 18G biopsy needle.

### Thoracic ultrasound protocol (Index test)

**Fig. 1:**
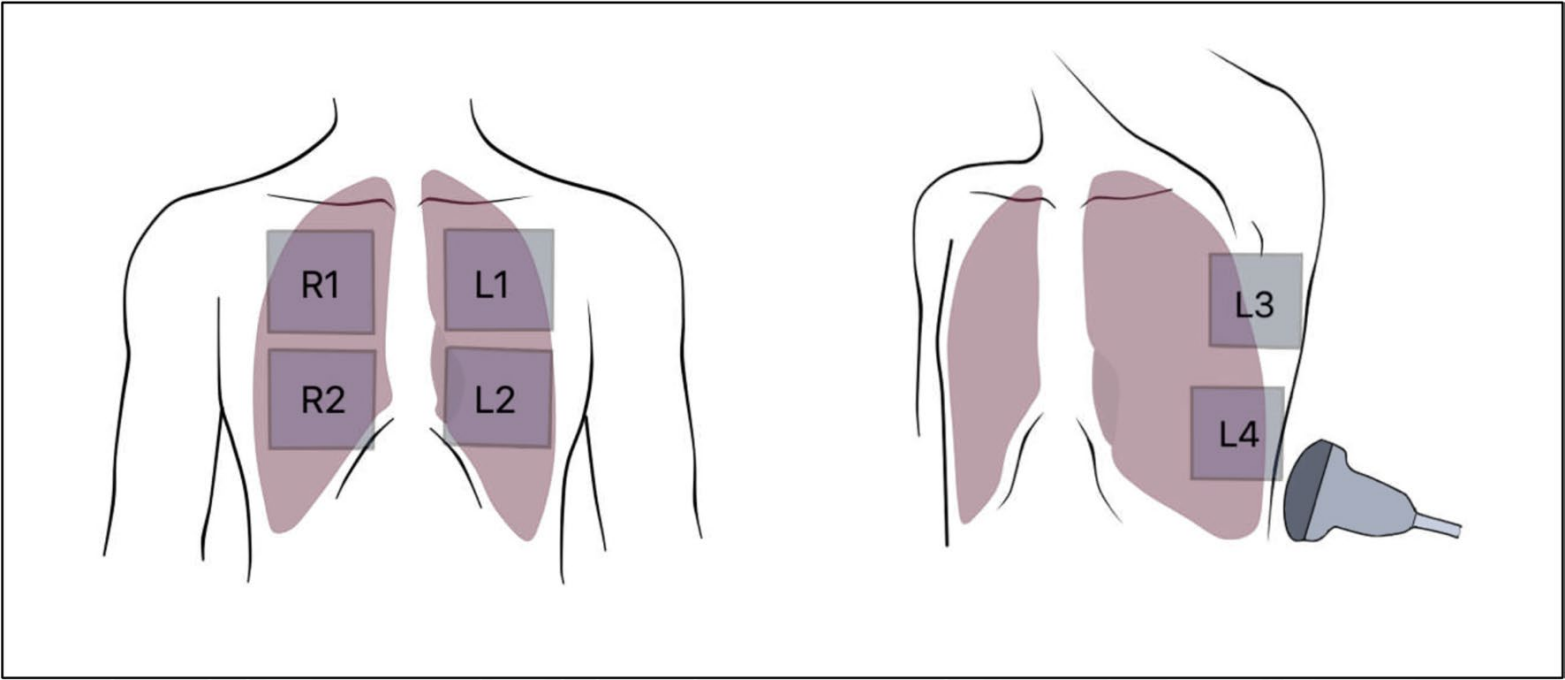
The zones scanned with Focused lung ultrasound (FLUS) protocol in this study—the illustrated zones 1–4 are scanned bilaterally (R = right, L = left), foregoing the commonly included three posterior zones on either side

**Fig. 2:**
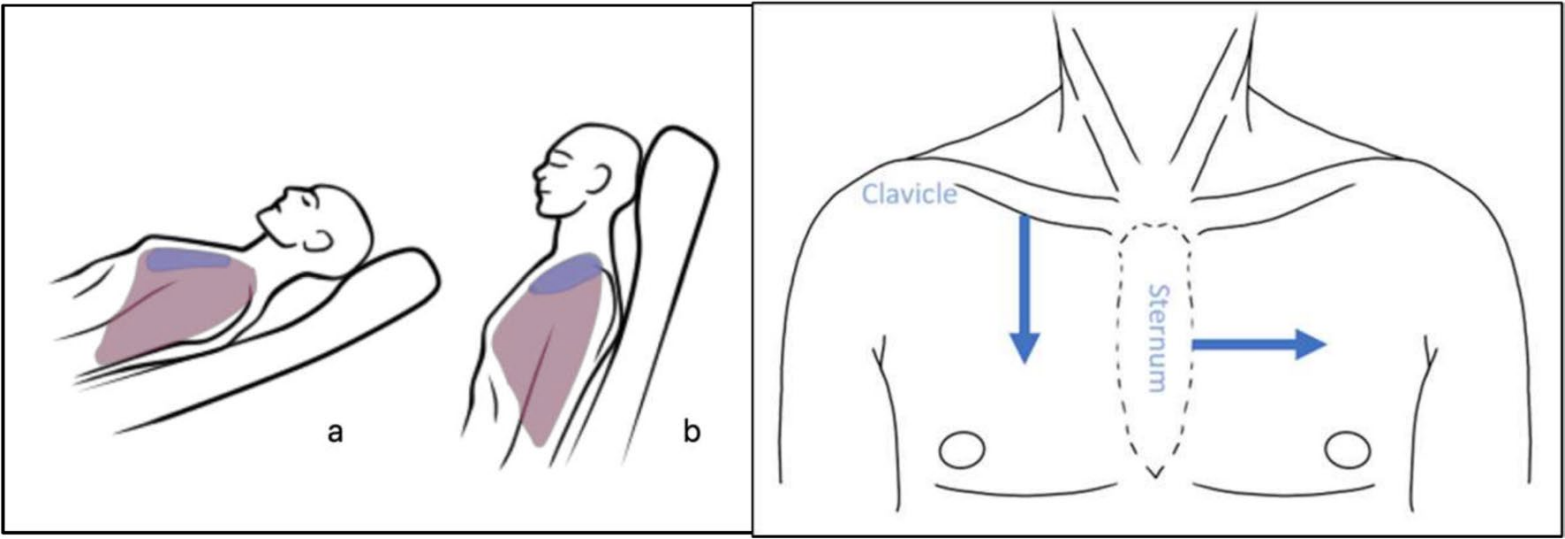
Placement of air in the pleural space in supine (**a**) and upright (**b**) position. Subsequently direction and measurement of the pneumothorax. In supine position from edge of sternum laterally to lung point. In an upright position from the edge of the clavicle caudally to lung point in the midclavicular line

Two medical students certified in Focused Lung Ultrasound (FLUS) performed the ultrasound scans and data collection using a national certification programme with validated theoretical and practical competence assessments [20, 21]. The scans were performed using a Siemens ACUSON P500 Portable Ultrasound Device with a Siemens CH5-2 abdominal probe (Siemens Healthineers AG, München, Germany) using a lung-specific preset. The ultrasound examination was performed according to the FLUS protocol in zones R1-R4 and L1-L4 in both upright and sitting positions [8], see Fig. 1. The potential absence of lung sliding (advocating pneumothorax) was noted in each zone. If the absence of lung sliding was noted, the lung point was searched. The size of the possible pneumothorax was estimated by measuring the distance from the lung point to clavicle in upright position, and lung point to sternum when in supine position, due to the positional movement of potential pleural air, see Fig. 2. The measurement was done at the fingers’ width for the operator’s and patient’s convenience and later converted to cm.

**Fig. 3:**
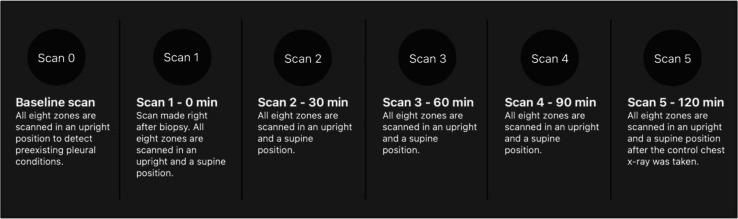
Timeline of FLUS examination of each patient

The ultrasound examination was performed six times, first as a baseline before the CT-guided transthoracic biopsy and subsequently five times after the procedure every 30 min (see Fig. 3). The last time was after the conventional 2-h chest X-ray control (standard-of-care in the institution). The results of the chest X-ray were blinded to the ultrasound operators.

The baseline scan was omitted if the patient’s preparation was delayed (n = 6 patients). Two patients experienced discomfort lying down, resulting in scans in an upright position only.

### Chest x-ray (Reference test)

The reference test was a conventional, standing chest x-ray in posterior-anterior and lateral projection. Following the chest x-ray, the radiographer in the observing room called a radiologist for assessment of the x-ray.

The scan results and relevant data from the patients’ electronic journal, including the chest X-ray results, were collected into the study’s RedCap database.

### Statistical analyses

All data extracted from patients’ electronic journals and the chest X-ray results were managed in RedCap [22]. The STATA 17 software (StataCorp, 2021, Stata Statistical Software: Release 17, College Station, TX, StataCorp, LLC) was used to perform the statistical analyses. No sample size was calculated before conducting the study due to the study design and time constraints. Missing data was left out of the statistical analysis. Descriptive statistics were performed for each variable. Categorical variables were presented with frequencies and percentages.

## Results

**Fig. 4:**
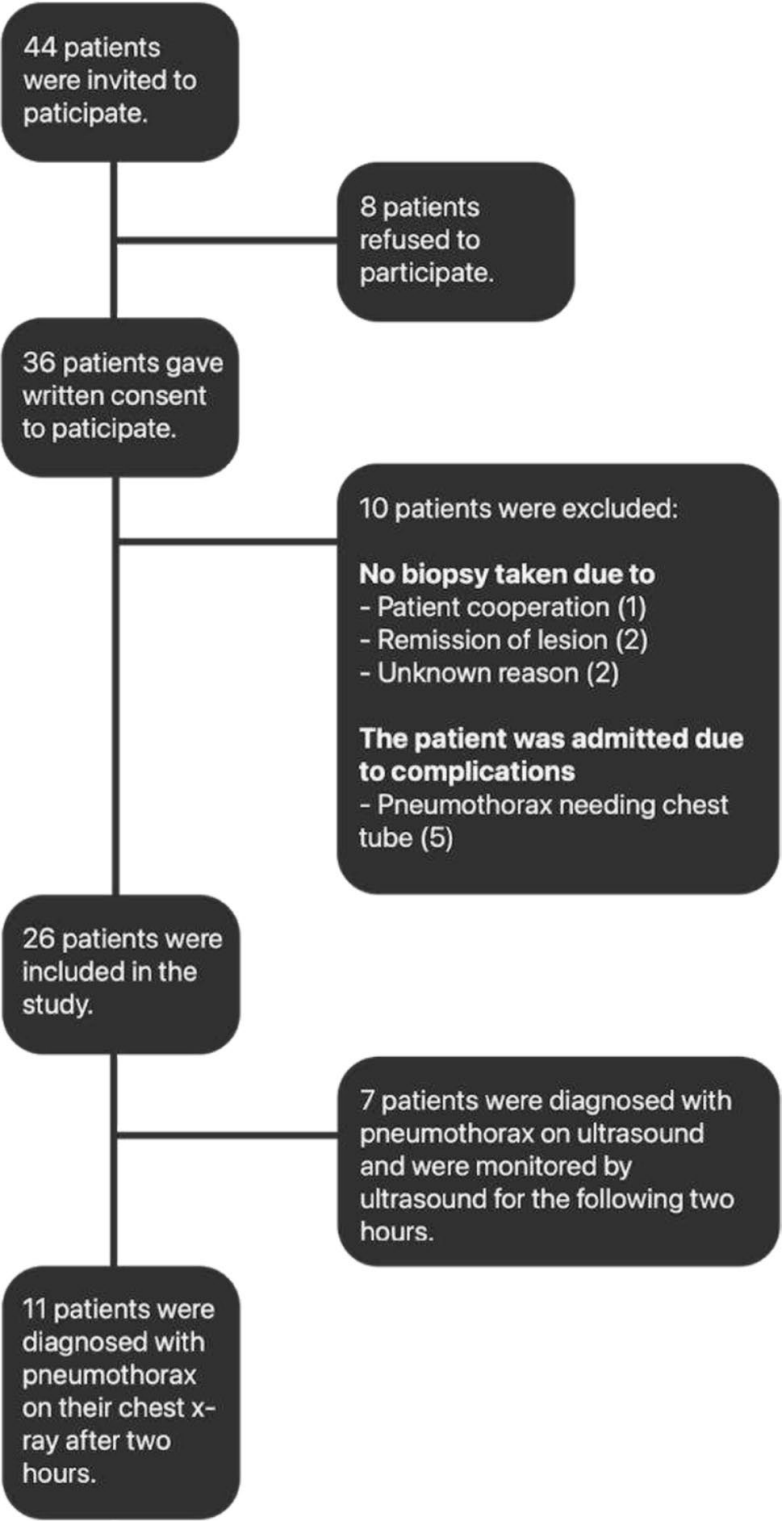
Patient flow chart, showing how patients were included in the study

A total of 44 patients were eligible for inclusion of which 26 were included in the study and received all five post-procedure ultrasound examinations, a patient flowchart is seen in Fig. 4. Patient demographics are presented in Table 1.

**Table 1:** Patient demographics

Characteristic	All patients (*n* = 26) Mean (SD) or *n* (%)	Patients with PTX (*n* = 11) Mean (SD) or *n* (%)
Age, years	71.5 (11.7)	72.7 (13.7)
Female	13 (50%)	5 (45,5%)
Male	13 (50%)	6 (54,5%)
BMI, kg/m^2^	25.5 (4.6)	24.6 (4.1)
Smoking status		
• Active	8 (30.8%)	5 (45.5%)
• Previous	15 (57.7%)	5 (45.5%)
• Non-smoker	2 (7.7%)	0 (0%)
• Missing data	1 (3.9%)	1 (9.1%)
Years of smoking	26.4 (18.5)	32.4 (18.6)
Tumor size, mm	26.7 (14.2)	22.4 (10.4)
Tumor location		
• Right upper lobe	4 (15.4%)	2 (18.2%)
• Right middle lobe	2 (7.7%)	0 (0%)
• Right lower lobe	9 (34.6%)	4 (36.4%)
• Left upper lobe	4 (15.4%)	1 (9.1%)
• Right lower lobe	7 (26.9%)	4 (36.4%)
Comorbidities		
• Previous PTX	1 (3.9%)	1 (9.1%)
• Previous cancer	11 (42.3%)	4 (36.4%)
• Other lung disease	1 (3.9%)	2 (18.2%)
• Hypertension	11 (42.3%)	3 (27.3%)
• Cardiac disease	10 (38.5%)	3 (27.3%)
• Diabetes Mellitus	3 (11.5%)	0 (0%)

Pneumothorax was diagnosed by standing posterior-anterior and lateral projection chest X-ray in 11 patients (42%). In comparison, pneumothorax was diagnosed by ultrasound in six patients in upright position and seven patients in supine position. From the data, sensitivity, specificity, positive predictive value and negative predictive value were calculated, showing best efficacy of the test when performed in supine position. All results can be seen in Tables 2, 3 and 4.

**Table 2:** Thoracic ultrasound diagnostic accuracy for pneumothorax in upright position, 2 × 2

Upright position				
		Chest X-ray 2 h post procedure		
		Positive	Negative	Total
Ultrasound examination	Positive	**7**	**1**	**8**
	Negative	**4**	**14**	**18**
	Total	**11**	**15**	**26**

**Table 3:** Thoracic ultrasound diagnostic accuracy for pneumothorax in supine position, 2 × 2

Supine position				
		Chest X-ray 2 h post procedure		
		Positive	Negative	Total
Ultrasound examination	Positive	**8**	**0**	**8**
	Negative	**3**	**15**	**18**
	Total	**11**	**15**	**26**

**Table 4:** Thoracic ultrasound diagnostic accuracy for pneumothorax

Ultrasound examination	Sensitivity	Specificity	Positive predictive value	Negative predictive value
Upright position	63.6%	93.3%	87.5%	77.8%
Supine position	72.3%	100%	100%	83.3%

One patient was excluded due to a technical error in evaluating the pneumothorax’s dynamic changes of over time. Overall, after the second ultrasound examination post-procedure (at 30 min), all the identified pneumothoraces were diagnosed by ultrasound.

**Fig. 5:**
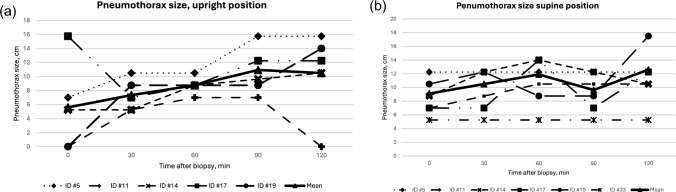
**a** Development of measured pneumothorax over time in an upright position. **b** Development of measured pneumothorax over time in supine position

The largest pneumothoraces in an upright position measured approximately 16 cm from the clavicle’s lower edge. They were diagnosed in two patients, one upon return from the biopsy and another patient at the last examination after the control chest x-ray. The largest measurement in the supine position was found in a patient at their last examination 2 h post-procedure, measuring 17.5 cm. The largest mean measurement occurred in the sitting position at 90 min and supine at 120 min: 12.6 cm and 10.9 cm, respectively. See Fig. 5a and b.

## Discussion

To our knowledge, this study is the first to investigate using ultrasound to detect *and* monitor pneumothoraces after CT-guided transthoracic biopsies. The study showed a moderate sensitivity and high specificity for ultrasound in detecting pneumothorax following CT-guided transthoracic lung biopsies. Even though, on average, most patients had minor sonographic progression of their pneumothoraces, the patients did not evolve symptoms needing intervention or admission during the observational period. It must be noted that patients with large pneumothoraces and/or severe symptoms were excluded from this study, creating a selection bias.

### Sensitivity and specificity

In our study population, 11 of 26 patients (42.3%) were diagnosed with pneumothorax. As mentioned, this was after the exclusion of the five patients diagnosed with large pneumothorax during the procedure who required direct admission to the respiratory department for drain insertion and closer monitoring than possible in the outpatient clinic in the radiological department. Thereby, the overall rate of pneumothorax in our population was a bit higher than current evidence, varying from as little as 2.8% [23] to up til 34.2% in other reviews and meta-analyses [3, 5, 6, 13, 24]. The occurrence rate of pneumothorax found in this study may be attributed to the risk factors associated with higher rates of pneumothorax in this specific population [4, 25, 26]. However, this study did not aim to explore or evaluate the risk factors and pneumothorax rates. With that said, the small dataset obtained during a shorter period of time in a still post-COVID era could theoretically not fully represent the true prevalence of pneumothorax in our population, and as known prevalence could affect positive predictive value and negative predictive value, but not sensitivity and specificity [27].

In studies involving populations similar to the one in our study, Satori et al. found both sensitivity and specificity of ultrasound to be 100% [23]. Hosseini-Nik found a sensitivity and specificity for ultrasound detection of small, as well as large pneumothoraces of 69.2% and 96%, respectively [24].

### Size monitoring

We managed to identify and monitor the small number of pneumothoraces in our study cohort, and we saw a minor increase in pneumothorax size during the observational period. However, none of the patients had significant symptoms or needed treatment during the observational period or the following days.

Hosseini-Nik et al. measured the size of the pneumothoraces by measuring how many of the first three intercostal spaces had ultrasonic signs of pneumothorax detected. They only did one ultrasound examination, whereas our study examines the development of pneumothoraces over time [24]. Sartori et al. repeated the ultrasound examination daily until the pneumothorax was resolved within 1–2 days but did not mention any findings or analysis on the size or development of pneumothoraces [23]. Thereby, there is still a lack of knowledge and evidence within this field of ultrasonic monitoring of post-procedural pneumothoraces but the study’s results are promising and we could potentially move towards more individual observational programs following transthoracic needle biopsies. Patients could be controlled and monitored using ultrasound and in the presence of pneumothorax more closely monitored. In our study, the patients with larger pneumothoraces were excluded. As newer guidelines on spontaneous pneumothorax suggest a more conservative and non-invasive approach [28], this could potentially also be the next step for the iatrogenic pneumothoraces and here ultrasound could play a crucial role with size monitoring.

### Positioning of the patients

Sartori et al. examined patients in supine and prone positions, and some were also examined sitting upright [23]. In contrast, Hosseini-Nik et al. had all patients scanned seated upright on a stretcher [24]. Air in the pleural space will, in most cases, move upwards, as shown in Fig. 2, meaning it would expectedly be more accessible to identify a pneumothorax in the supine position, as the structures overlying the lung apex could ‘hide’ a small pneumothorax on FLUS in an upright position. This is in line with the findings of our study, as our data shows that some pneumothoraces can be found in the supine position despite being undetectable in the upright position. Pneumothorax progression was mostly seen from 60–90 min following the procedure, for future studies and potential implementation of ultrasound detection of pneumothoraces, the ultrasound operator must reflect on the most optimal position with the best ultrasound view of the pleura before 60 min.

### Ultrasound operator

The two ultrasound operators were certified in a national simulation-based training programme including a theoretical and practical test, but did not have significant clinical lung ultrasound experience. When ultrasound trainees have reached sufficient fundamental knowledge and skills in a training programme hosted in a simulation-based setting, it is important to transfer the skills sufficiently into a clinical setting. However, the transfer and integration of skills into clinical practice is a process that needs supervision and sparring with other colleagues [29]. Many studies do not mention the number of operators performing ultrasound examinations in diagnostic accuracy studies or their field of expertise or their experience. This is very likely to influence the efficacy of ultrasound as a test for diagnosing pneumothorax [10, 11]. In the study by Hosseini-Nik et al., one radiologist with five years of experience performed the ultrasound examination [24]. Sartori et al. and Laursen et al. used an experienced physician for all ultrasound examinations in their studies [18, 23]. Using one exceptionally experienced physician could harm the before-mentioned studies’ external validity, as in a clinical setting, it is doubtful that it will always be the same, highly experienced physician performing all the tests and it is well known that sensitivity and specificity of a test often decrease when moving from the diagnostic accuracy study into a clinical setting. In our study, there was probably an unknown effect from certified though clinically inexperienced ultrasound operators that could potentially impact the efficacy of our test.

### Strengths and limitations

As previously mentioned, more studies suggest that ultrasound is superior in diagnosing pneumothorax compared to chest X-ray [10, 11]. However, when exploring the diagnostic accuracy of ultrasound in this setting it would have increased the level of evidence if chest CT was used as the gold standard, which unfortunately was not possible in our setting.

The small data set of only 26 patients from one hospital entails a low external validity but, to the best of our knowledge, no other studies have monitored post-CT-guided transthoracic biopsy patients that close using ultrasound, which require a set-up with one ultrasound operator in the observational room all the time conducting ultrasound examinations very frequently in the patients. The tendency of the results discovered in could indicate a relevant clinical impact and more studies with larger data sets should be conducted for results to be generalized to larger populations and to finally confirm the value of ultrasound in this setting.

### Future perspectives

From a future perspective, in a clinical setting, one or two scans in a supine position post-procedure would likely be enough to make the diagnosis, as pneumothorax was diagnosed by or before the scan at 30 min in supine position on all seven patients where pneumothorax was diagnosed with ultrasound. If explored further and proven consistent, this could mean discharging patients without ultrasonic signs of pneumothorax earlier and potentially sparing time for both patients and personnel. Subsequently,

The in-depth study design including a total of 11 ultrasound examinations (baseline, five uprights and five supines) per patient within two hours of the procedure requires significant time and resources, which must be considered in case of upscaling, and one could argue to scan only in a supine position. One study shows that an ultrasound examination, on average, is faster than a chest X-ray [10]. If the observation personnel, in our case radiographers, were trained in using ultrasound to detect pneumothoraces, it would be quicker than bringing the patient to a chest X-ray in another room or another part of the radiological department.

*In conclusion,* the results of this study suggest that ultrasound is feasible after CT-guided transthoracic lung biopsy to diagnose and monitor pneumothorax. The study showed that ultrasound as a diagnostic test in the supine position had a moderate sensitivity and high specificity in the detection of pneumothorax in this setting. All pneumothoraxes that were identified by ultrasound were detected within the first 30 min post-procedure. The dynamic changes of pneumothoraxes were investigated, showing that pneumothorax size did not increase to a level where the patient needed admittance. These results are not conclusive due to the small sample size but can be used as a base for the possibility and reasonability of reproducing similar studies in larger populations. Especially regarding the dynamic change of pneumothorax over time since few large studies have explored this.

## Data Availability

Data is available on request.

## Abbreviations

CT: Computed tomography
FLUS: Focused lung ultrasound
